# 2450 Delirium and catatonia: Age matters

**DOI:** 10.1017/cts.2018.159

**Published:** 2018-11-21

**Authors:** Jo E. Wilson, Richard Carlson, Maria C. Duggan, Pratik Pandharipande, Timothy D. Girard, Li Wang, Jennifer L. Thompson, Rameela Chandrasekhar, Andrew Francis, Stephen E. Nicolson, Robert S. Dittus, Stephan Heckers, E. W. Ely

**Affiliations:** 1 Department of Psychiatry, Vanderbilt University Medical Center, Nashville, TN, USA; 2 Department of Medicine, Division of General Internal Medicine and Public Health, Vanderbilt University School of Medicine, Nashville, TN, USA; 3 Veteran’s Affairs TN Valley, Geriatrics Research, Education and Clinical Center (GRECC); 4 Department of Anesthesiology and Critical Care; 5 Department of Critical Care Medicine, Clinical Research, Investigation, and Systems Modeling of Acute illness (CRISMA) Center, University of Pittsburgh School of Medicine, Pittsburgh, PA, USA; 6 Department of Biostatistics, Vanderbilt University Medical Center, Nashville, TN, USA; 7 Department of Psychiatry, Penn State Medical School, Hershey Medical Center, Hershey, PA, USA; 8 Department of Psychiatry, Beth Israel Deaconess Hospital-Plymouth, Plymouth, MA, USA; 9 Department of Medicine, The Center for Health Services Research, Division of Pulmonary and Critical Care, Vanderbilt University Medical Center, Nashville, TN, USA

## Abstract

OBJECTIVES/SPECIFIC AIMS: Background: Delirium is a well described form of acute brain organ dysfunction characterized by decreased or increased movement, changes in attention and concentration as well as perceptual disturbances (i.e., hallucinations) and delusions. Catatonia, a neuropsychiatric syndrome traditionally described in patients with severe psychiatric illness, can present as phenotypically similar to delirium and is characterized by increased, decreased and/or abnormal movements, staring, rigidity, and mutism. Delirium and catatonia can co-occur in the setting of medical illness, but no studies have explored this relationship by age. Our objective was to assess whether advancing age and the presence of catatonia are associated with delirium. METHODS/STUDY POPULATION: Methods: We prospectively enrolled critically ill patients at a single institution who were on a ventilator or in shock and evaluated them daily for delirium using the Confusion Assessment for the ICU and for catatonia using the Bush Francis Catatonia Rating Scale. Measures of association (OR) were assessed with a simple logistic regression model with catatonia as the independent variable and delirium as the dependent variable. Effect measure modification by age was assessed using a Likelihood ratio test. RESULTS/ANTICIPATED RESULTS: Results: We enrolled 136 medical and surgical critically ill patients with 452 matched (concomitant) delirium and catatonia assessments. Median age was 59 years (IQR: 52–68). In our cohort of 136 patients, 58 patients (43%) had delirium only, 4 (3%) had catatonia only, 42 (31%) had both delirium and catatonia, and 32 (24%) had neither. Age was significantly associated with prevalent delirium (i.e., increasing age associated with decreased risk for delirium) (*p*=0.04) after adjusting for catatonia severity. Catatonia was significantly associated with prevalent delirium (*p*<0.0001) after adjusting for age. Peak delirium risk was for patients aged 55 years with 3 or more catatonic signs, who had 53.4 times the odds of delirium (95% CI: 16.06, 176.75) than those with no catatonic signs. Patients 70 years and older with 3 or more catatonia features had half this risk. DISCUSSION/SIGNIFICANCE OF IMPACT: Conclusions: Catatonia is significantly associated with prevalent delirium even after controlling for age. These data support an inverted U-shape risk of delirium after adjusting for catatonia. This relationship and its clinical ramifications need to be examined in a larger sample, including patients with dementia. Additionally, we need to assess which acute brain syndrome (delirium or catatonia) develops first.

